# Association of Prehospital Oxygen Saturation to Inspired Oxygen Ratio With 1-, 2-, and 7-Day Mortality

**DOI:** 10.1001/jamanetworkopen.2021.5700

**Published:** 2021-04-13

**Authors:** Francisco Martín-Rodríguez, Raúl López-Izquierdo, Carlos del Pozo Vegas, Juan F. Delgado-Benito, Guillermo J. Ortega, Miguel A. Castro Villamor, Ancor Sanz-García

**Affiliations:** 1 Faculty of Medicine, Valladolid University, Valladolid, Spain; 2 Advanced Life Support, Emergency Medical Services, Valladolid, Spain; 3 Emergency Department, Hospital Universitario Rio Hortega, Valladolid, Spain; 4 Emergency Department, Hospital Clínico Universitario, Valladolid, Spain; 5 Data Analysis Unit, Health Research Institute, Hospital de la Princesa, Madrid, Spain; 6 Consejo Nacional de Investigaciones Científicas y Técnicas, Buenos Aires, Argentina

## Abstract

**Importance:**

The early identification of patients at high risk of clinical deterioration represents one of the greatest challenges for emergency medical services (EMS).

**Objective:**

To assess whether use of the ratio of prehospital oxygen saturation measured by pulse oximetry (Spo_2_) to fraction of inspired oxygen (Fio_2_) measured during initial contact by EMS with the patient (ie, the first Spo_2_ to Fio_2_ ratio) and 5 minutes before the patient’s arrival at the hospital (ie, the second Spo_2_ to Fio_2_ ratio) can predict the risk of early in-hospital deterioration.

**Design, Setting, and Participants:**

A prospective, derivation-validation prognostic cohort study of 3606 adults with acute diseases referred to 5 tertiary care hospitals in Spain was conducted between October 26, 2018, and June 30, 2020. Eligible patients were recruited from among all telephone requests for EMS assistance for adults who were later evacuated with priority in advanced life support units to the referral hospitals during the study period.

**Main Outcomes and Measures:**

The primary outcome was hospital mortality from any cause within the first, second, third, or seventh day after EMS transport to the hospital. The main measure was the Spo_2_ to Fio_2_ ratio.

**Results:**

A total of 3606 participants comprised 2 separate cohorts: the derivation cohort (3081 patients) and the validation cohort (525 patients). The median age was 69 years (interquartile range, 54-81 years), and 2122 patients (58.8%) were men. The overall mortality rate of the patients in the study cohort ranged from 3.6% for 1-day mortality (131 patients) to 7.1% for 7-day mortality (256 patients). The best model performance was for 2-day mortality with the second Spo_2_ to Fio_2_ ratio with an area under the curve of 0.890 (95% CI, 0.829-0.950; *P* < .001), although the other outcomes also presented good results. In addition, a risk-stratification model was generated. The optimal cutoff resulted in the following ranges of Spo_2_ to Fio_2_ ratios: 50 to 100 for high risk of mortality, 101 to 426 for intermediate risk, and 427 to 476 for low risk.

**Conclusions and Relevance:**

This study suggests that use of the prehospital Spo_2_ to Fio_2_ ratio was associated with improved management of patients with acute disease because it accurately predicts short-term mortality.

## Key Points

**Question:**

Can the prehospital ratio of oxygen saturation measured by pulse oximetry (Spo_2_) to fraction of inspired oxygen (Fio_2_) be used to model early in-hospital mortality?

**Findings:**

Data from 3606 patients were analyzed in a prognostic study. The Spo_2_ to Fio_2_ ratio had a statistically significant area under the curve of 0.890 for the prediction of 2-day mortality.

**Meaning:**

This study suggests that the prehospital Spo_2_ to Fio_2_ ratio allows for the identification of patients at risk of in-hospital deterioration.

## Teaser

This prognostic study assesses whether use of the ratio of prehospital oxygen saturation to fraction of inspired oxygen measured by emergency medical services can predict the risk of early in-hospital deterioration.

## Introduction

Emergency medical services (EMS) not only represent the initial contact between the patient and the health system, but it is also usually the gateway for the patient to the emergency department.^1,2^ New prehospital care procedures include different tools, such as the use of early warning scores (eg, the National Early Warning Score, the VitalPAC Early Warning Score, and the Modified Early Warning Score),^3,4^ which have the fundamental challenge of detecting patients at high risk of clinical deterioration. Oxygen saturation as measured by pulse oximetry (Spo_2_) is present in almost all the scores.

The use of Spo_2_ is a routine, noninvasive, continuous, and safe standard procedure implemented in most multiparameter monitors in prehospital care, which, together with the use of capnography, can help to determine the patient’s ventilatory status more precisely.^5,6^ In addition, as of the initial contact between EMS health care workers and the patient, the fraction of inspired oxygen (Fio_2_) is known precisely. The combined use of both parameters, known as the Spo_2_ to Fio_2_ ratio, has demonstrated its clinical utility in the context of hospital care, particularly in intensive care units, for patients receiving noninvasive or invasive mechanical ventilation, even though it is not routinely used in prehospital care.^7,8^

Although the use of partial pressure of oxygen in arterial blood (Pao_2_) has been shown to be a very robust clinical indicator,^9,10^ many patients do not undergo an arterial blood gas analysis in prehospital scenarios because obtaining arterial blood samples and having point-of-care testing are not generalized procedures in ambulances. Instead, the Spo_2_ to Fio_2_ ratio can be an alternative for noninvasive and continuous ventilatory function monitoring; several studies have analyzed the use of the Spo_2_ to Fio_2_ ratio as a reliable proxy of the Pao_2_to Fio_2_ ratio.^11,12,13^

In the prehospital scenario, the patient’s history is sometimes unknown, symptoms are diffuse, and response time must be rapid; in addition, decisions regarding treatment and possible referral to the emergency department are based on the results of on-site clinical examination, standard vital signs, and an electrocardiogram.^14^ Emergency medical services personnel must have diagnostic strategies to identify patients with hidden acute respiratory failure or hypoxemia, which are sometimes not clearly identified and are detected only with subsequent studies in the emergency department.^15,16^

We therefore investigated the performance of the Spo_2_ to Fio_2_ ratio, both during initial contact between EMS personnel in the ambulance and the patient (ie, the first Spo_2_ to Fio_2_ ratio) and 5 minutes before the patient’s arrival at the hospital (ie, the second Spo_2_ to Fio_2_ ratio) to identify the risk of early in-hospital deterioration, including mortality within 1, 2, 3, and 7 days after the index event, in people with acute diseases treated by EMS.

## Methods

### Study Design

We conducted a prospective, multicenter, EMS delivery, ambulance-based, derivation-validation, prognostic cohort study of adults (>18 years of age) with acute diseases. Patients were referred with high priority by the advanced life support units to 5 tertiary care hospitals of the public health system of Castile and Leon, Spain, with a reference population of 1 364 952 inhabitants. Data came from 2 studies conducted under the same procedure but during different periods: the derivation cohort (ISRCTN17676798), obtained between October 26, 2018, and October 31, 2019, and the validation cohort (ISRCTN48326533), obtained between January 1 and June 30, 2020. The advanced life support team includes a physician, an emergency registered nurse, and 2 paramedics with specific training, operating in nonstop mode (24 hours per day and 7 days per week), performing standard life support maneuvers on the scene and en route, according to protocols. The study protocol was approved by the local institutional research review boards of Complejo Asistencial de Segovia, Hospital Universitario de Burgos, Complejo Asistencial Universitario de Salamanca, Hospital Clínico Universitario de Valladolid, and Hospital Universitario Rio Hortega de Valladolid. Patients or legal guardians provided written informed consent. This study is reported according to the Strengthening the Reporting of Observational Studies in Epidemiology (STROBE) reporting guideline.

### Study Population

Eligible patients were recruited from among all telephone requests for emergency assistance from adults who were later evacuated with priority by advanced life support to the referral hospitals during the study period. Exclusion criteria were cardiorespiratory arrest, terminal illness, pregnancy, patients evacuated by other means of transport (eg, basic life support), and cases in which, after evaluation by the physician, the patient was discharged in situ. Cases in which health care workers were in jeopardy (eg, assault, stabbing, gun shot, or hazardous material) were not evaluated for eligibility. Patients for whom it was impossible to calculate Spo_2_ to Fio_2_ ratios because of the lack of some needed variable were also excluded.

During prehospital care and when the patient’s clinical situation allowed, the patient or a legal guardian read and signed the informed consent that covered the whole study. The emergency registered nurse managed the primary consent process. In cases in which it was impossible to obtain consent on the scene or en route, the associate coordinator of each hospital was responsible for obtaining informed consent. All patients without informed consent were excluded.

### Outcome

The primary outcome was in-hospital cumulative mortality from any cause within the first, second, third, or seventh day after EMS transport to the hospital. Patients included in previous time points for mortality were also considered for the next time point for mortality (eg, mortality on the second day also included patients who died during the first day). The final result of death was recorded in each hospital by the research coordinator, based on the review of the patient’s electronic medical record.

### Predictors and Data Abstraction

During initial contact with the patient, the emergency registered nurse recorded family status, age, sex, and intervention times as well as the set of vital signs (respiratory rate and Spo_2_) and basal Fio_2_ in the standardized clinical history used by EMS professionals. The Spo_2_ and Fio_2_ were recorded at 2 times: just after the ambulance arrived at the scene (ie, during initial evaluation) and 5 minutes before the patient arrived at the hospital and after prehospital ventilatory support for those who needed it. With these 2 time points, respectively, the first Spo_2_ to Fio_2_ ratio and the second Spo_2_ to Fio_2_ ratio were subsequently calculated. The Spo_2_ was measured using the LIFEPAK 15 defibrillator monitor (Physio-Control Inc) with Masimo rainbow technology (Masimo). Both the first Spo_2_ to Fio_2_ ratio and the second Spo_2_ to Fio_2_ ratio were the only variables included in the prediction model.

The physician recorded any type of ventilatory support (nasal cannula, nebulizer, Venturi mask, reservoir masks, noninvasive mechanical ventilation, and invasive mechanical ventilation) used at the scene or en route and diagnosed the corresponding group of symptoms according to the *International Classification of Diseases, 11th Revision*. Seven days after the index event, the hospital outcomes were obtained by reviewing the electronic medical record: inpatients, intensive care unit admissions, and mortality within 1, 2, 3, and 7 days. To guarantee the traceability of the data, the exact link was made by matching 5 of 6 extractors: date, family status, age, sex, admission time, and personal health care card number. The statistical power calculation can be found in the eAppendix in the Supplement.

### Statistical Analysis

**Figure 1.**
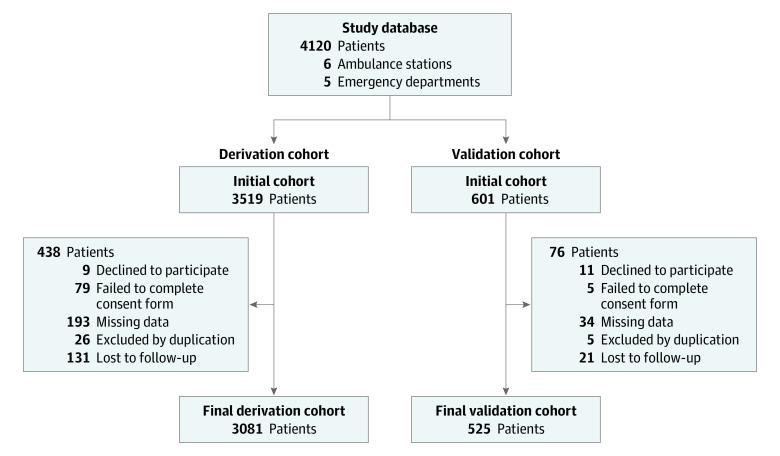
Flowchart Showing the Analysis Population

All patient data were recorded electronically in a database created specifically for this purpose. The case registration form was tested to eliminate ambiguous elements and to validate the data collection instrument. Patients with missing data were excluded (Figure 1).

Normality tests were performed on all the quantitative variables (Shapiro-Wilk and Lilliefors tests). Quantitative variables were described as median and interquartile range (25th-75th percentile). The categorical variables were described using absolute frequencies and percentages.

For the comparison of the mean values of the quantitative variables, the Mann-Whitney test was used; the χ^2^ test was used for 2 × 2 contingency tables to assess the association between qualitative variables. The Fisher exact test was used when it was necessary.

The discriminative power of the predictor variable was performed through a prediction model using a generalized linear model. The model included the outcome variable and the predictor variable. The prediction model was built using the derivation cohort. To assess the validity of the model for predicting mortality, we determined the area under the curve (AUC) of the receiver operating characteristic of the model in the validation cohort. The *P* value of the hypothesis test (null hypothesis: AUC = .50) and its corresponding 95% CI were also assessed. Further statistical characteristics, such as the positive predictive value, negative predictive value, positive likelihood ratio, negative likelihood ratio, odds ratio, and diagnostic accuracy, were determined.

**Figure 2.**
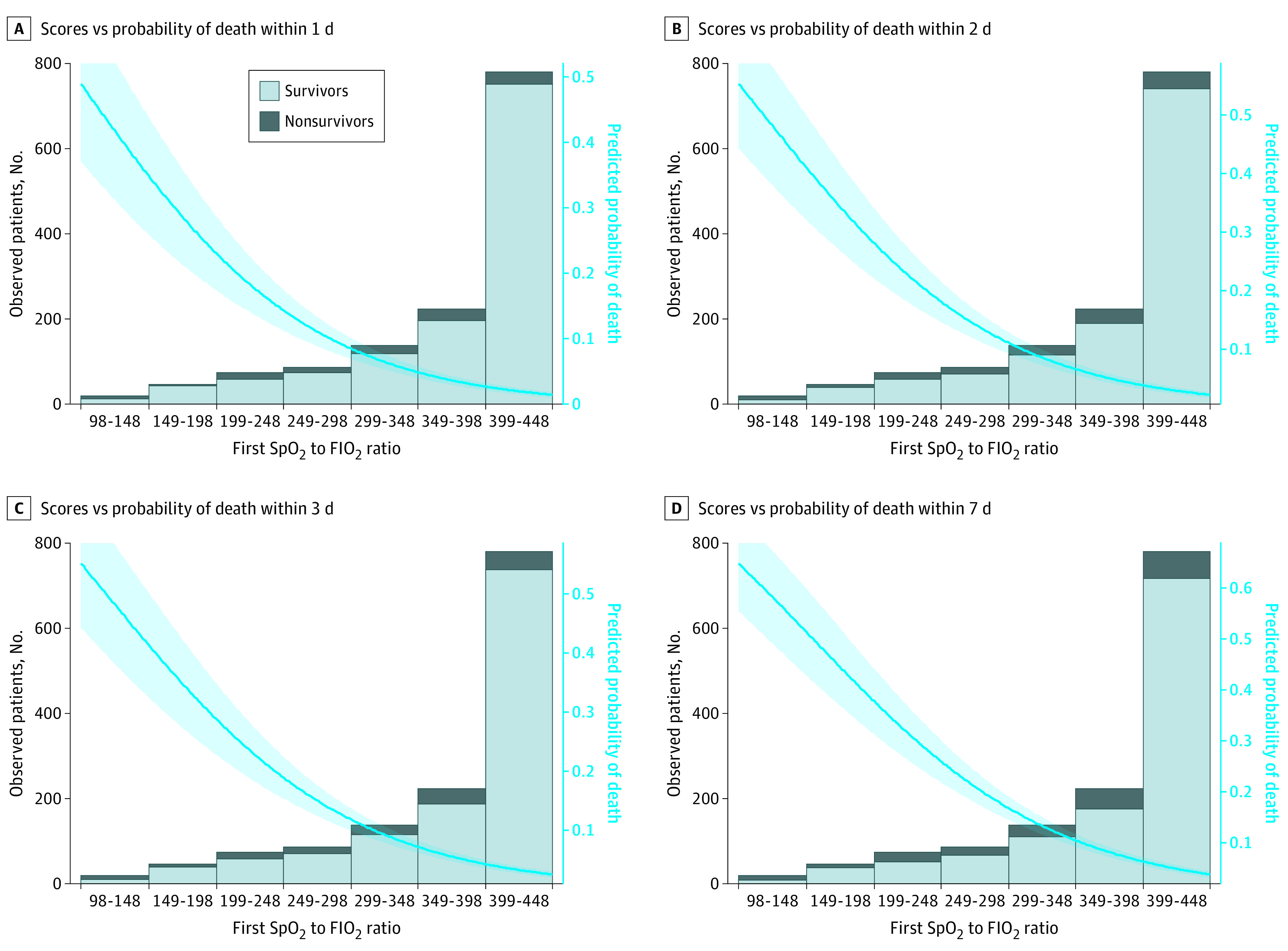
First Spo_2_ to Fio_2_ Ratio Scores vs Real and Predicted Probability of Death A, Scores vs probability of death within 1 day. B, Scores vs probability of death within 2 days. C, Scores vs probability of death within 3 days. D, Scores vs probability of death within 7 days. The shaded area outside the trend line corresponds to the 95% CI of the predicted probability of death. Fio_2_ indicates fraction of inspired oxygen; Spo_2_, oxygen saturation as measured by pulse oximetry.

**Figure 3.**
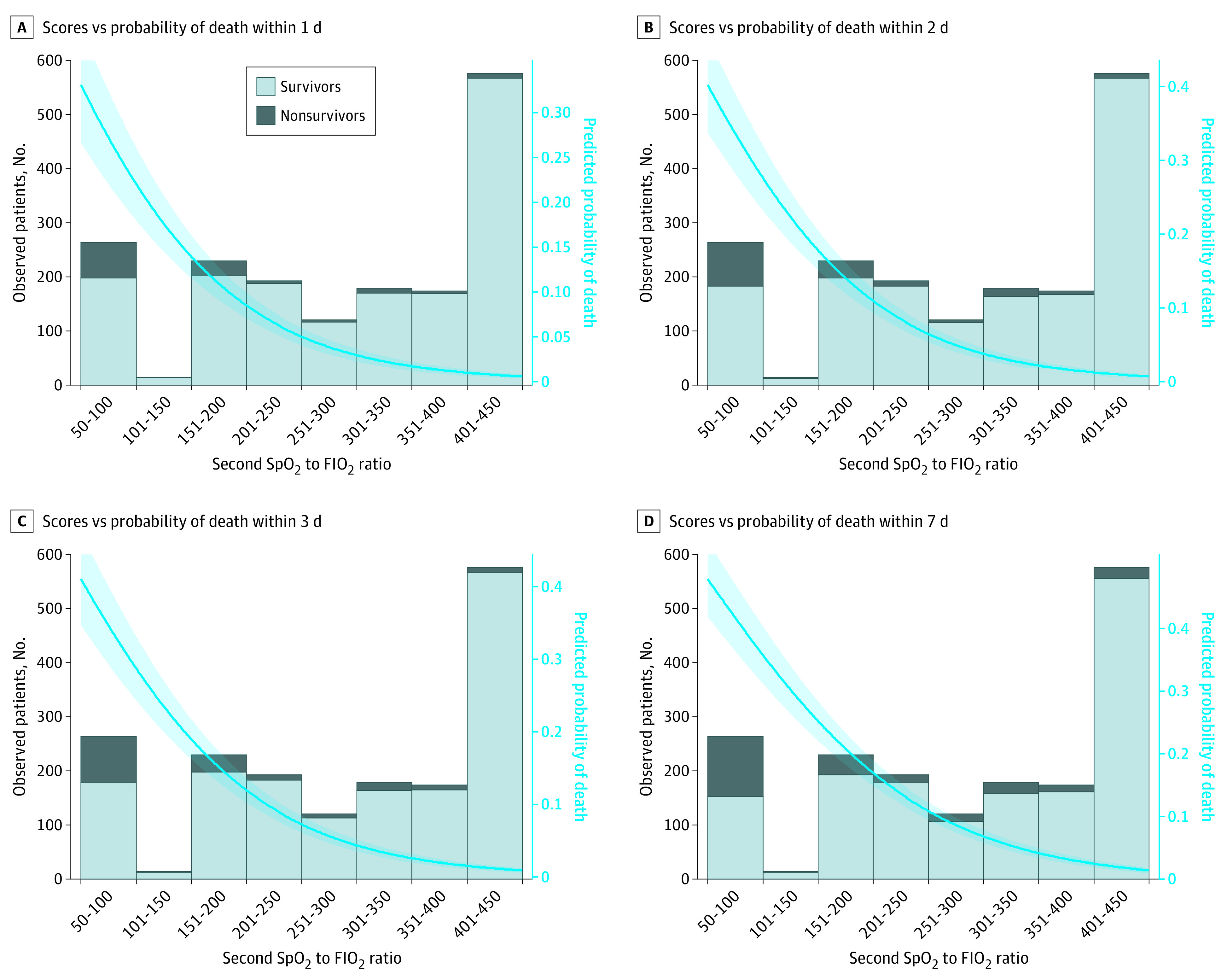
Second Spo_2_ to Fio_2_ Ratio Scores vs Real and Predicted Probability of Death A, Scores vs probability of death within 1 day. B, Scores vs probability of death within 2 days. C, Scores vs probability of death within 3 days. D, Scores vs probability of death within 7 days. The shaded area outside the trend line corresponds to the 95% CI of the predicted probability of death. Fio_2_ indicates fraction of inspired oxygen; Spo_2_, oxygen saturation as measured by pulse oximetry.

One of the most valuable characteristics of a predictive model is its capacity to classify patients according to their mortality risk. In this sense, we considered 3 categories (high, intermediate, and low mortality risk). The category cutoff points were derived from the graphical representation of the first Spo_2_ to Fio_2_ ratio or the second Spo_2_ to Fio_2_ ratio, according to the predicted probability of death (Figure 2 and Figure 3); in particular, ranges were selected as follows: Spo_2_ to Fio_2_ ratios between minimum and minimum plus 50 correspond to a low risk, Spo_2_ to Fio_2_ ratios between minimum plus 50 and maximum minus 50 correspond to an intermediate risk, and Spo_2_ to Fio_2_ ratios between maximum minus 50 and maximum correspond to a high risk.

In addition, the discrimination capacity for the best model was assessed considering confounding factors (sex and age). In other words, the AUC of the receiver operating characteristic was determined for each category of these variables; for example, for sex, the cohort resulting from adding both cohorts was split into female and male, and the AUC was calculated for each new data set. All statistical analyses were performed using our own codes and base functions in R, version 3.5.1 (R Foundation for Statistical Computing).^17^ All *P* values were from 2-sided tests and results were deemed statistically significant at *P* < .05.

## Results

### Patient Characteristics

During the study period, 4119 patients were examined for eligibility, based on 6 ambulance stations and transport to the emergency departments of 5 public hospitals. For the analysis, 3606 patients were enrolled: 3081 patients in the derivation cohort and 525 in the validation cohort (Figure 1). The median age was 69 years (interquartile range, 54-81 years), 2122 patients (58.8%) were men, and 1484 patients (41.2%) were women. Demographic characteristics and clinical data are described in Table 1.

**Table 1.** Baseline Characteristics

Characteristic	No. (%) (N = 3606)
Age, median (IQR), y	69 (54-81)
Age group, y	
18-49	697 (19.3)
50-60	580 (16.1)
61-75	992 (27.5)
76-85	845 (23.4)
>85	492 (13.6)
Sex	
Female	1484 (41.2)
Male	2122 (58.8)
Self-reported race/ethnicity	
White	3566 (98.9)
Afro-Europeans	29 (0.8)
Asian	2 (0.06)
Multiple	9 (0.2)
Isochronous time, median (IQR), min	
Arrival	10 (8-14)
Support	28 (22-35)
Evacuation	10 (7-14)
Total time	50 (42-60)
Basal evaluation	
Breathing rate, median (IQR), breaths/min	18 (14-23)
Spo_2_, median (IQR), %	96 (93-98)
Supplemental O_2_	440 (12.2)
Fio_2_, median (IQR), %	0.21 (0.21-0.21)
First Spo_2_ to Fio_2_ ratio, median (IQR)	457 (438-467)
Prehospital ventilatory support	
Nasal cannula	370 (10.3)
Nebulizer	338 (9.4)
Venturi mask	218 (6.0)
Reservoir masks	229 (6.4)
NIMV	93 (2.6)
IMV	165 (4.6)
Pretransfer evaluation	
Breathing rate, median (IQR), breaths/min	15 (12-19)
Spo_2_, median (IQR), %	96 (94-98)
Supplemental O_2_	
Fio_2_, median (IQR), %	0.21 (0.21-0.28)
Second Spo_2_ to Fio_2_ ratio, median (IQR)	452 (336-467)
Hospital outcomes	
Inpatients	2050 (56.8)
ICU	317 (8.8)
1-d Mortality	131 (3.6)
2-d Mortality	166 (4.6)
3-d Mortality	180 (5.0)
7-d Mortality	256 (7.1)
Pathologic conditions	
Infectious	253 (7.0)
Neurological	664 (18.4)
Cardiovascular	1487 (41.2)
Respiratory	322 (8.9)
Digestive	189 (5.2)
Trauma and external agents	426 (11.8)
Poisoning	210 (5.8)
Other^a^	55 (1.5)

Abbreviations: Fio_2_, fraction of inspired oxygen; ICU, intensive care unit; IMV, invasive mechanical ventilation; IQR, interquartile range; NIMV, noninvasive mechanical ventilation; Spo_2_, oxygen saturation measured by pulse oximetry.

^a^ Other pathologic conditions: endocrine, genitourinary and diseases of the blood and the immune system.

The differences between survivors and nonsurvivors were significant in both the first Spo_2_ to Fio_2_ ratio (survivors, 452; and nonsurvivors, 160; *P* < .001) and the second Spo_2_ to Fio_2_ ratio (survivors, 452; and nonsurvivors, 166; *P* < .001). A comparison between survivors and nonsurvivors can be found in eTable 1 and eTable 2 in the Supplement.

### Mortality Outcomes

The overall mortality rate ranged from 3.6% (131 patients) for 1-day mortality (49.6% [65 of 131] of intensive care unit admissions) to 7.1% (256 patients) for 7-day mortality (47.3% [121 of 256] of intensive care unit admissions). Cardiovascular diseases represented the highest percentage of 7-day mortality (86 [33.6%]), followed by neurologic and infectious pathologic conditions (Table 1).

In terms of mortality, patients with early mortality had lower Spo_2_ to Fio_2_ ratios than patients with later mortality. In the case of the first Spo_2_ to Fio_2_ ratio, this trend was also observed in Spo_2_ with constant values of Fio_2_, but in the case of the second Spo_2_ to Fio_2_ ratio, prehospital intervention, usually with ventilatory support, improved Spo_2_ at the expense of an increase in Fio_2_.

### Validity of the Spo_2_ to Fio_2_ Ratio

The first Spo_2_ to Fio_2_ ratio showed the best predictive capacity for 2-day mortality, with an AUC of 0.810 (95% CI, 0.739-0.881), even though all outcomes presented similar AUC values of 0.798 (95% CI, 0.721-0.874) for 1-day mortality, 0.805 (95% CI, 0.737-0.873) for 3-day mortality, and 0.779 (95% CI, 0.711-0.847) for 7-day mortality (all *P* < .001) (eFigure 1 in the Supplement). The mortality distribution according to the first Spo_2_ to Fio_2_ ratio and the predicted probability of mortality is shown in Figure 2.

The same procedure was used to assess the validity of the second Spo_2_ to Fio_2_ ratio. The best performance was again obtained for 2-day mortality, with an AUC of 0.890 (95% CI, 0.829-0.950). The other outcomes presented similar AUC values: 0.876 (95% CI, 0.803-0.948) for 1-day mortality, 0.877 (95% CI, 0.817-0.937) for 3-day mortality, and 0.857 (95% CI, 0.797-0.916) for 7-day mortality (*P* < .001 for all cases) (eFigure 2 in the Supplement). The mortality distribution according to the second Spo_2_ to Fio_2_ ratio and the predicted probability of mortality is shown in Figure 3. Further statistical details of the models are shown in eTable 3 in the Supplement.

Table 2 shows the percentages of mortality for the 3 mortality risk categories of the first and second Spo_2_ to Fio_2_ ratios. The optimal cutoff resulted in the following ranges of Spo_2_ to Fio_2_ ratios: 50 to 100 for high risk of mortality, 101 to 426 for intermediate risk, and 427 to 476 for low risk.

**Table 2.** Mortality Rate for the 3 Mortality Risk Categories for the First and Second Spo_2_ to Fio_2_ Ratios

Mortality	High risk (range, 50-100)		Intermediate risk (range, 101-426)		Low risk (range, 427-476)	
	Survivors	Nonsurvivors	Survivors	Nonsurvivors	Survivors	Nonsurvivors
First Spo_2_ to Fio_2_ ratio (basal assessment)^a^						
At 1 d	11/17 (64.7)	6/17 (35.2)	647/734 (88.1)	87/734 (11.9)	2816/2854 (98.6)	38/2854 (1.4)
At 2 d	9/17 (52.9)	8/17 (47.1)	627/734 (85.4)	107/734 (14.6)	2803/2854 (98.2)	51/2854 (1,8)
At 3 d	9/17 (52.9)	8/17 (47.1)	622/734 (84.7)	112/734 (15.3)	2794/2854 (97.8)	60/2854 (2.2)
At 7 d	8/17 (47.1)	9/17 (52.9)	586/734 (79.9)	148/734 (20.1)	2756/2854 (96.5)	98/2854 (3.5)
Second Spo_2_ to Fio_2_ ratio (after prehospital ventilatory support)^a^						
At 1 d	196/262 (74.8)	66/262 (25.2)	951/1004 (94.7)	53/1004 (5.3)	2327/2339 (99.5)	12/2339 (0.5)
At 2 d	181/262 (69.1)	81/262 (30.9)	933/1004 (92.9)	71/1004 (7.1)	2325/2339 (99.4)	14/2339 (0.6)
At 3 d	176/262 (67.1)	86/262 (32.9)	928/1004 (92.4)	76/1004 (7.6)	2321/2339 (99.2)	18/2339 (0.8)
At 7 d	151/262 (57.6)	111/262 (42.4)	899/1004 (89.5)	105/1004 (10.5)	2299/2339 (98.2)	40/2339 (1.8)

Abbreviations: Fio_2_, fraction of inspired oxygen; Spo_2_, oxygen saturation measured by pulse oximetry.

^a^ Values expressed as total number (fraction).

Finally, to rule out the association of confounding factors in the predictive capacity of the model, the AUC of the receiver operating characteristic for 2-day mortality of the second Spo_2_ to Fio_2_ ratio was assessed for sex and age. The AUC was 0.862 (95% CI, 0.817-0.907; *P* < .001) for women and 0.888 (95% CI, 0.855-0.921; *P* < .001) for men. Ages were categorized into 5 ranges: 18 to 49 years (AUC, 0.983 [95% CI, 0.969-0.996]; *P* < .001), 50 to 60 years (AUC, 0.974 [95% CI, 0.957-0.991]; *P* < .001), 61 to 75 years (AUC, 0.866 [95% CI, 0.800-0.932]; *P* < .001), 76 to 85 years (AUC, 0.840 [95% CI, 0.778-0.902]; *P* < .001), and older than 85 years (AUC, 0.792 [95% CI, 0.730-0.853]; *P* < .001).

Additional time points for mortality were considered (6-hour and 30-day mortality for the first Spo_2_ to Fio_2_ ratio); both were associated with lower AUC values (6-hour mortality: AUC, 0.769 [95% CI, 0.654-0.883]; 30-day mortality: AUC, 0.786 [95% CI, 0.725-0.846]) compared with the prior results. Last, the predictive validity of Spo_2_ was evaluated for 1-day mortality, yielding a lower AUC compared with the Spo_2_ to Fio_2_ ratio (0.782 [95% CI, 0.688-0.877]).

## Discussion

To our knowledge, this is the first prospective, multicenter, EMS delivery, ambulance-based, derivation-validation, prognostic cohort study of adults that evaluates the capacity of prehospital Spo_2_ to Fio_2_ ratios to predict the clinical risk of in-hospital deterioration, including mortality within 1, 2, 3, and 7 days after the index event. Both the first Spo_2_ to Fio_2_ ratio (basal assessment) and the second Spo_2_ to Fio_2_ ratio (just before hospital admission) presented a good prognostic validity to predict the risk of early mortality, with better results for the second Spo_2_ to Fio_2_ ratio, particularly for 2-day mortality.

Our study demonstrates that the Spo_2_ to Fio_2_ ratio may be a putative marker of prehospital acute disease–associated mortality. The determination of this parameter provides relevant information on respiratory function, which seems to be associated with a short- and medium-term poor prognosis.

The Spo_2_ to Fio_2_ ratio is used in clinical practice in cases of acute respiratory distress syndrome,^18^ for control during noninvasive mechanical ventilation,^19^ as a proxy measure for the calculation of the sepsis-related organ failure assessment score when Pao_2_ is not available,^20^ or, more recently, for continuous monitoring of ventilatory function in patients with COVID-19.^21^ Outside the hospital context, Batchinsky et al^22^ evaluated the capacity of the Spo_2_ to Fio_2_ ratio as a surrogate of the Pao_2_ to Fio_2_ ratio for 30 anesthetized swine in a simulated altitude situation with few changes of having bedside point-of-care testing and requiring complex evacuation procedures; the authors concluded that the Spo_2_ to Fio_2_ ratio may be used as a reliable substitute for the Pao_2_to Fio_2_ ratio.

Ambulance staff are trained to perform advanced airway support, frequently using devices and techniques in the prehospital setting that were formerly used exclusively in hospitals, such as invasive mechanical ventilation (through orotracheal intubation and video laryngoscopes) and the increasingly used noninvasive mechanical ventilation.^23,24^ Patients who require advanced procedures of airway management should be accompanied by continuous monitoring, using Spo_2_ as standard to monitor oxygenation.^25^ In addition, capnography (end-tidal Co_2_) can be used to assess ventilation,^5,6^ which is also useful for predicting mortality.^26^

Both the current clinical validity of the Spo_2_ to Fio_2_ ratio and the most frequent advanced airway support performed in prehospital care suggest the importance of the Spo_2_ to Fio_2_ ratio as a good candidate for prediction of outcomes. The present Spo_2_ to Fio_2_ ratios classified patients into 3 groups by stratifying the risk of deterioration. A high level of vigilance should be maintained for patients at intermediate risk (Spo_2_ to Fio_2_ ratio, 101-426) and high risk (Spo_2_ to Fio_2_ ratio, 50-100). Low-risk patients (Spo_2_ to Fio_2_ ratio, 427-476) had a low probability of clinical deterioration (1.8% [51 of 2854] in the worst case of the second Spo_2_ to Fio_2_ ratio); in this last case, however, these results do not completely exclude the patient’s clinical deterioration or the appearance of an acute disease, but it helps EMS personnel in performing a more precise initial evaluation. The early identification of high-risk patients in the prehospital setting is a main goal of EMS that can help improve the management of these patients.^27,28^

The EMS scenario is complex, and personnel should make decisions quickly and with a limited number of complementary tests,^29^ so any potentially helpful diagnostic and/or prognostic tool must be seriously considered. Among the great variability of pathologic conditions, comorbidities, and risky situations present in prehospital settings, time-dependent pathologic conditions should be effectively discriminated from pathologic conditions that, although potentially serious, may involve a greater delay in caring or evacuation.^30^ In these situations, the early warning scores and biomarkers serve to standardize decision-making for EMS professionals.^3,31^ In this context, the Spo_2_ to Fio_2_ ratio provides a simple, continuous, and noninvasive monitoring tool applicable in any clinical situation capable of being handled even by personnel with little training. The Spo_2_ to Fio_2_ ratio also provides the extra advantage of providing a simple stratification of the patient’s risk of clinical deterioration. This discrimination between patients at intermediate and high risk allows for appropriate treatments or surveillance measures to be implemented.

The second Spo_2_ to Fio_2_ ratio has a better predictive capacity for mortality than the baseline Spo_2_ to Fio_2_ ratio, an issue that leads us to stress the importance of continuous monitoring. This will offer real-time information on respiratory support.

### Limitations

Our study has several limitations. First, our end point was in-hospital mortality from any cause within 7 days after the initial care, ruling out out-of-hospital mortality within the selected period. The objective of this study was to evaluate the association of the acute disease leading to prehospital activation with the in-hospital short-term outcome. Future studies will consider the comorbidities and the evolution in the medium to long term. Second, a patient selection bias exists because the sample was recruited using the opportunity criteria during the study period, including only patients evaluated and evacuated by advanced life support units. To minimize bias, the study involved units working in rural and urban areas, during all time points and during the 4 seasons. Despite this, the final sample comprised a very high percentage of elderly adults with outcomes in line with similar studies.^32,33^ Third, the data extractors were not blinded. To ensure that the outcomes were not subject to interpretation, a double-check (by an associate researcher from each hospital and the principal investigator) was performed on cases that presented mortality within 7 days of care.

## Conclusions

Both the first Spo_2_ to Fio_2_ ratio (basal assessment) and the second Spo_2_ to Fio_2_ ratio (before hospital admission) presented particularly good prognostic capacities to predict the risk of in-hospital clinical deterioration within 7 days of hospital admission. The Spo_2_ to Fio_2_ ratio is a useful substitute for the Pao_2_ to Fio_2_ ratio, to be used in prehospital care for the early detection of patients at high risk of clinical deterioration. The standardized use of early warning scores, biomarkers, or any diagnostic or prognostic tool that could help in the complex decision-making process must be considered and implemented in EMS procedures.

## Notes

Supplement. **eTable 1.** Baseline Characteristics, Mortality Rates for One and Two Day **eTable 2.** Baseline Characteristics, Mortality Rates for Three and Seven Day **eTable 3.** Statistical Details of the Models for SaFi 1 (Basal Assessment) and SaFi 2 (After Prehospital Ventilatory Support) **eFigure 1.** Receiver Operational Curve (ROC) of SaFi 1 for the Different Outcomes: One (a), Two (b), Three (c), and Seven (d) Day Mortality **eFigure 2.** Receiver Operational Curve (ROC) of SaFi 2 for the Different Outcomes: One (a), Two (b), Three (c), and Seven (d) Day Mortality **eAppendix.** Statistical Power Calculation
