# Supplementary material for: Association of Prehospital Oxygen Saturation to Inspired Oxygen Ratio With 1-, 2-, and 7-Day Mortality
Source: JAMA Netw Open. 2021 Apr 13;4(4):e215700. doi: 10.1001/jamanetworkopen.2021.5700 (PMC8044733; doi:10.1001/jamanetworkopen.2021.5700)
Supplement: Supplement. — eTable 1. Baseline Characteristics, Mortality Rates for One and Two Day eTable 2. Baseline Characteristics, Mortality Rates for Three and Seven Day eTable 3. Statistical Details of the Models for SaFi 1 (Basal Assessment) and SaFi 2 (After Prehospital Ventilatory Support) eFigure 1. Receiver Operational Curve (ROC) of SaFi 1 for the Different Outcomes: One (a), Two (b), Three (c), and Seven (d) Day Mortality eFigure 2. Receiver Operational Curve (ROC) of SaFi 2 for the Different Outcomes: One (a), Two (b), Three (c), and Seven (d) Day Mortality eAppendix. Statistical Power Calculation [file jamanetwopen-e215700-s001.pdf]

# Supplementary Online Content

Martín-Rodríguez F, López-Izquierdo R, del Pozo Vegas C, et al. Association of prehospital oxygen saturation to inspired oxygen ratio with 1-, 2-, and 7-day mortality. *JAMA Netw Open.* 2021;4(4):e215700. doi:10.1001/jamanetworkopen.2021.5700

**eTable 1.** Baseline Characteristics, Mortality Rates for One and Two Day
**eTable 2.** Baseline Characteristics, Mortality Rates for Three and Seven Day
**eTable 3.** Statistical Details of the Models for SaFi 1 (Basal Assessment) and SaFi 2 (After Prehospital Ventilatory Support)
**eFigure 1.** Receiver Operational Curve (ROC) of SaFi 1 for the Different Outcomes: One (a), Two (b), Three (c), and Seven (d) Day Mortality
**eFigure 2.** Receiver Operational Curve (ROC) of SaFi 2 for the Different Outcomes: One (a), Two (b), Three (c), and Seven (d) Day Mortality
**eAppendix.** Statistical Power Calculation

This supplementary material has been provided by the authors to give readers additional information about their work.

**eTable 1.** Baseline Characteristics, Mortality Rates for One and Two Day

| Characteristics[1] | 1-day Survivors | Non-survivors | *P* value[2] | 2-day Survivors | Non-survivors | *P* value[2] |
|---|---|---|---|---|---|---|
| No. of patients | 3475 | 131 | | 3440 | 166 | |
| Age, median, years | 69 (53-81) | 80 (66-87) | .000 | 69 (53-81) | 79 (66-87) | .000 |
| Sex, female, No. (%) | 1432 (41.2) | 52 (39.7) | .730 | 1420 (41.3) | 64 (38.6) | .486 |
| Basal evaluation, median | | | | | | |
| Breathing rate, breaths/min | 18 (14-22) | 24 (11-34) | .000 | 17 (14-22) | 24 (14-34) | .000 |
| $SpO_2$, % | 96 (93-98) | 82 (72-93) | .000 | 96 (93-98) | 84 (73-93) | .000 |
| Supplemental $O_2$, No. (%) | 397 (11.4) | 43 (32.8) | .000 | 386 (11.2) | 54 (32.5) | .000 |
| FiO2, % | 0.21 (0.21-0.21) | 0.21 (0.21-0.24) | .000 | 0.21 (0.21-0.21) | 0.21 (0.21-0.24) | .000 |
| SaFi 1 | 457 (438-467) | 367 (286-438) | .000 | 457 (438-467) | 375 (288-438) | .000 |
| SBP, mmHg | 138 (120-156) | 120 (87-148) | .000 | 138 (120-156) | 124 (91-150) | .000 |
| DBP, mmHg | 80 (68-91) | 68 (48-89) | .000 | 80 (68-91) | 71 (49-90) | .000 |
| Heart rate, beats/min | 84 (70-103) | 97 (74-122) | .008 | 84 (70-103) | 97 (74-122) | .000 |
| Temperature, ºC | 36.3 (36-36.8) | 36 (35.1-37) | .020 | 36.3 (36-36.8) | 36 (35.1-37) | .024 |
| GCS, points | 15 (15-15) | 11 (4-15) | .000 | 15 (15-15) | 10 (4-15) | .000 |
| Pre-transfer evaluation, median | | | | | | |
| Breathing rate, breaths/min | 15 (12-18) | 19 (15-28) | .000 | 14 (12-18) | 17 (15-28) | .000 |
| $SpO_2$, % | 97 (94-98) | 92 (90-97) | .000 | 97 (94-98) | 92 (90-97) | .000 |
| FiO2, % | 0.21 (0.21-0.28) | 0.5 (0.35-0.99) | .000 | 0.21 (0.21-0.26) | 0.5 (0.31-0.99) | .000 |
| SaFi 2 | 452 (350-467) | 160 (92-269) | .000 | 452 (354-467) | 166 (92 - 295) | .000 |
| SBP, mmHg | 130 (113-147) | 97 (76-131) | .000 | 130 (113-147) | 101 (78-132) | .000 |
| DBP, mmHg | 73 (64-83) | 57 (45-73) | .000 | 73 (64-83) | 60 (45-75) | .000 |
| Heart rate, beats/min | 80 (68-96) | 92 (67-115) | .001 | 80 (68-96) | 93 (67-114) | .000 |
| Temperature, ºC | 36.1 (35.8-36.6) | 36 (34.9-36.7) | .421 | 36.1 (35.8-36.6) | 36 (35-36.7) | .397 |
| GCS, points | 15 (15-15) | 9 (3-14) | .000 | 15 (15-15) | 8 (3-15) | .000 |
| Hospital outcomes | | | | | | |
| Inpatients, No. (%) | 1919 (55.2) | 131 (100) | .000 | 1884 (54.8) | 166 (100) | .000 |
| ICU, No. (%) | 252 (7.3) | 65 (49.6) | .000 | 232 (6.7) | 85 (51.2) | .000 |

$SpO_2$: pulse oximetry saturation; $FiO_2$: fraction of inspired oxygen; SaFi: pulse oximetry saturation/fraction of inspired oxygen; SBP: systolic blood pressure; DBP: diastolic blood pressure; GCS: Glasgow coma scale

[1]Values expressed as total number (fraction) and medians [25 percentile-75 percentile], as appropriate

[2]*p* value were calculated using a $X^2$ test, *t* test, or Wilcoxon.

© 2021 Martín-Rodríguez F et al. *JAMA Network Open.*

**eTable 2.** Baseline Characteristics, Mortality Rates for Three and Seven Day

| Characteristics[1] | 3-day Survivors | Non-survivors | *P* value[2] | 7-day Survivors | Non-survivors | *P* value[2] |
|---|---|---|---|---|---|---|
| No. of patients | 3426 | 180 | | 3350 | 256 | |
| Age, median, years | 69 (53-81) | 79 (67-87) | .000 | 68 (53-81) | 80 (68-87) | .000 |
| Sex, female, No. (%) | 1416 (41.3) | 68 (37.8) | .345 | 1381 (41.2) | 103 (40.2) | .757 |
| Basal evaluation, median | | | | | | |
| Breathing rate, breaths/min | 17 (14-22) | 24 814-34) | .000 | 17 (14-22) | 21 (14-32) | .000 |
| $SpO_2$, % | 96 (93-98) | 85 (74-93) | .000 | 96 (94-98) | 88 (75-95) | .000 |
| Supplemental $O_2$, No. (%) | 385 (11.2) | 55 (30.6) | .000 | 367 (11) | 73 (28.5) | .000 |
| FiO2, % | 0.21 (0.21-0.21) | 0.21 (0.21-0.24) | .000 | 0.21 (0.21-0.21) | 0.21 (0.21-0.24) | .000 |
| SaFi 1 | 457 (443-467) | 381 (301-438) | .000 | 457 (443-467) | 383 (306-448) | .000 |
| SBP, mmHg | 138 (120-156) | 126 (94-154) | .001 | 138 (110-156) | 132 (88-155) | .000 |
| DBP, mmHg | 80 (68-91) | 73 (50-90) | .000 | 80 (69-91) | 73 (56-90) | .000 |
| Heart rate, beats/min | 84 (70-103) | 95 (70-120) | .009 | 84 (70-102) | 92 (74-120) | .001 |
| Temperature, ºC | 36.3 (36-36.8) | 36.1 (35.3-37) | .020 | 36.3 (36-36.8) | 36.1 (35.5-36.9) | .019 |
| GCS, points | 15 (15-15) | 10 (4-15) | .000 | 15 (15-15) | 11 (5-15) | .000 |
| Pre-transfer evaluation, median | | | | | | |
| Breathing rate, breaths/min | 14 (12-18) | 16 (15-28) | .000 | 14 812-18) | 17 (15-26) | .000 |
| $SpO_2$, % | 97 (94-98) | 92 (90-97) | .000 | 97 (94-98) | 93 (90-97) | .000 |
| FiO2, % | 0.21 (0.21-0.26) | 0.5 (0.28-0.99) | .000 | 0.21 (0.21-0.26) | 0.5 (0.28-0.99) | .000 |
| SaFi 2 | 452 (357-467) | 172 (92-314) | .000 | 452 (370-467) | 182 (94-349) | .000 |
| SBP, mmHg | 130 (113-147) | 103 (78-133) | .000 | 130 (113-147) | 110 (86-138) | .000 |
| DBP, mmHg | 73 (64-83) | 60 (45-75) | .000 | 73 (64.83) | 60 (48-76) | .000 |
| Heart rate, beats/min | 80 (68-96) | 90 (47-113) | .002 | 80 (65-85) | 88 (68-110) | .000 |
| Temperature, ºC | 36.1 (35.8-36.6) | 36 (35-36.7) | .386 | 36.1 (35.9-36.6) | 36 (35.1-36.7) | .732 |
| GCS, points | 15 (15-15) | 8 (3-15) | .000 | 15 815-15) | 10 (3-15) | .000 |
| Hospital outcomes | | | | | | |
| Inpatients, No. (%) | 1870 (54.6) | 180 (100) | .000 | 1794 (53.6) | 256 (100) | .000 |
| ICU, No. (%) | 224 (6.5) | 93 (51.7) | .000 | 196 (5.9) | 121 (47.3) | .000 |

$SpO_2$: pulse oximetry saturation; $FiO_2$: fraction of inspired oxygen; SaFi: pulse oximetry saturation/fraction of inspired oxygen; SBP: systolic blood pressure; DBP: diastolic blood pressure; GCS: Glasgow coma scale

[1]Values expressed as total number (fraction) and medians [25 percentile-75 percentile], as appropriate

system

[2]*p* value were calculated using a $X^2$ test, *t* test, or Wilcoxon.

© 2021 Martín-Rodríguez F et al. *JAMA Network Open.*

**eTable 3.** Statistical Details of the Models for SaFi 1 (Basal Assessment) and SaFi 2 (After Prehospital Ventilatory Support)

| | | Mortality rates[1] | | | |
|---|---|---|---|---|---|
| | | 1-day | 2-days | 3-days | 7-days |
| SaFi 1 | | | | | |
| | Sensitivity | 33.9 (31.0-36.8) | 32.8 (29.9-35.6) | 31.4 (28.7 (34.2) | 29.6 (27.0-32.3) |
| | Specificity | 89.0 (87.3-90.8) | 89.2 (87.5-91.0) | 89.2 (87.5-91.0) | 89.6 (87.8-91.3) |
| | PPV | 20.3 (18.9-21.6) | 23.8 (22.4-25.2) | 24.2 (22.8-25.5) | 32.6 (30.9-34.3) |
| | NPV | 97.4 (97.3-97.5) | 96.6 (96.5-96.8) | 96.3 (96.2-96.4) | 94.5 (94.4-94.7) |
| | Likelihood ratio (+) | 8.2 (7.3-9.2) | 7.8 (7.0-8.6) | 7.2 (6.5-7.9) | 8.5 (7.2-9.03) |
| | Likelihood ratio (-) | 0,70 (0.67-0.72) | 0.71 (0.68-0.73) | 0.72 (0.70-0.75) | 0.75 (0.72.0.77) |
| | Youden Point | 440.4 | 440.4 | 440.4 | 440.4 |
| | Odds ratio | 0.98 (0.98-0.99) | 0.98 (0.98-0.99) | 0.98 (0.98-0.99) | 0.98 (0.98-0.99) |
| SaFi 2 | | | | | |
| | Sensitivity | 66.8 (64.4-69.3) | 66.5 (64.0-69.0) | 64.9 (62.5-67.4) | 60.0 (57.7-62.4) |
| | Specificity | 80.1 (78.5-81.8) | 80.6 (78.9-82.3) | 80.7 (79.1-82.4) | 81.4 (79.7-83.1) |
| | PPV | 15.2 (14.4-15.9) | 18.7 (17.8-19.6) | 19.9 (18.9-20.8) | 25.3 (24.2-26.5) |
| | NPV | 98.6 (98.5-98.7) | 98.2 (98.1-98.3) | 97.9 (97.8-98.1) | 96.6 (96.5-96.8) |
| | Likelihood ratio (+) | 5.1 (4.7-5.3) | 5.1 (4.8-5.4) | 5.1 (4.8-5.4) | 4.9 (4.6-5.2) |
| | Likelihood ratio (-) | 0.37 (0.35-0.39) | 0.37 (0.35-0.39) | 0.39 (0.37-0.41) | 0.45 (0.43-0.47) |
| | Youden Point | 231.2 | 383.9 | 383.9 | 383.9 |
| | Odds ratio | 0.98 (0.98-0.99) | 0.98 (0.98-0.99) | 0.98 (0.98-0.99) | 0.98 (0.98-0.99) |

SaFi: pulse oximetry saturation / fraction of inspired oxygen; PPV: positive predictive value; NPV: negative predictive value

[1]Bracketed numbers indicate 95% confidence interval

© 2021 Martín-Rodríguez F et al. *JAMA Network Open.*

**eFigure 1.** Receiver Operational Curve (ROC) of SaFi 1 for the Different Outcomes: One (a), Two (b), Three (c), and Seven (d) Day Mortality

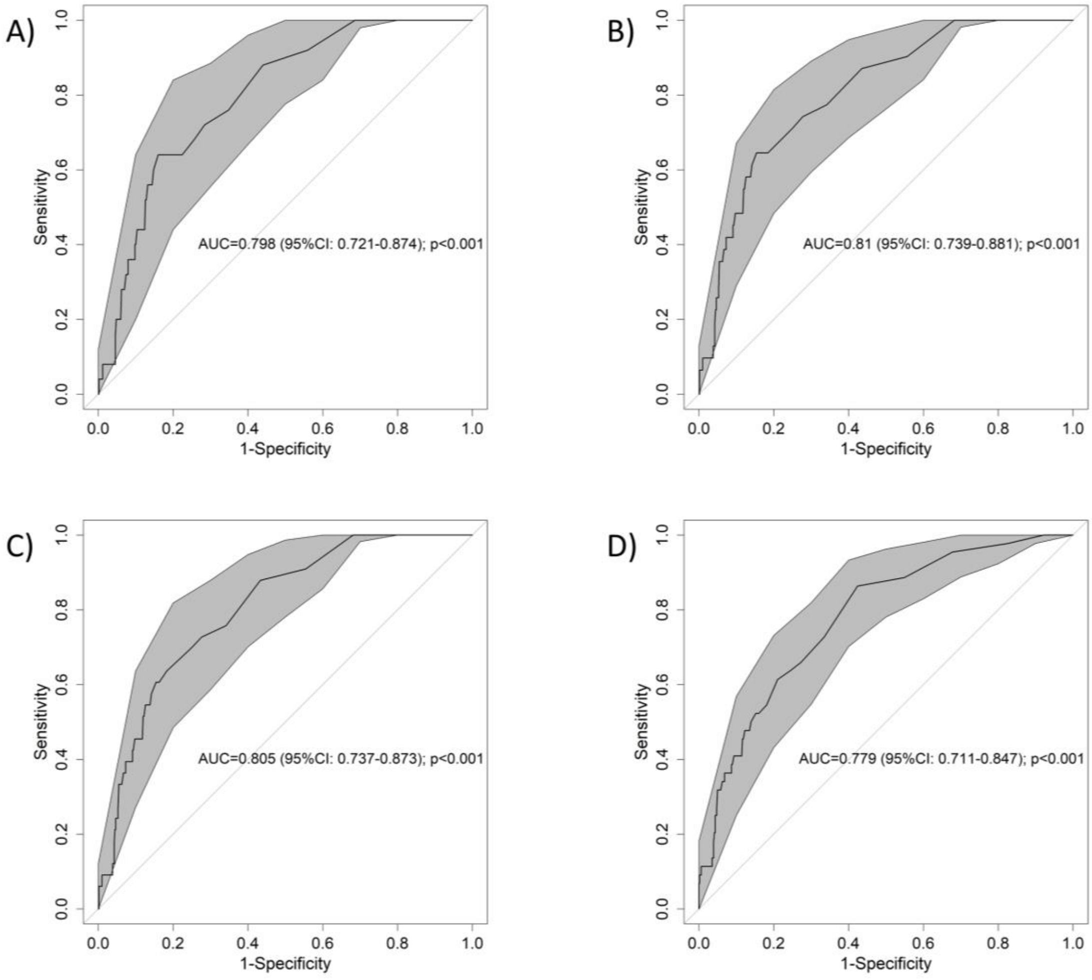


The bold line shows the ROC curve value, and the grey shading is the result of the validation coefficient. In the center of the graph is the area under the curve (AUC) and its 95% confidence interval.

© 2021 Martín-Rodríguez F et al. *JAMA Network Open.*

**eFigure 2.** Receiver Operational Curve (ROC) of SaFi 2 for the Different Outcomes: One (a), Two (b), Three (c), and Seven (d) Day Mortality

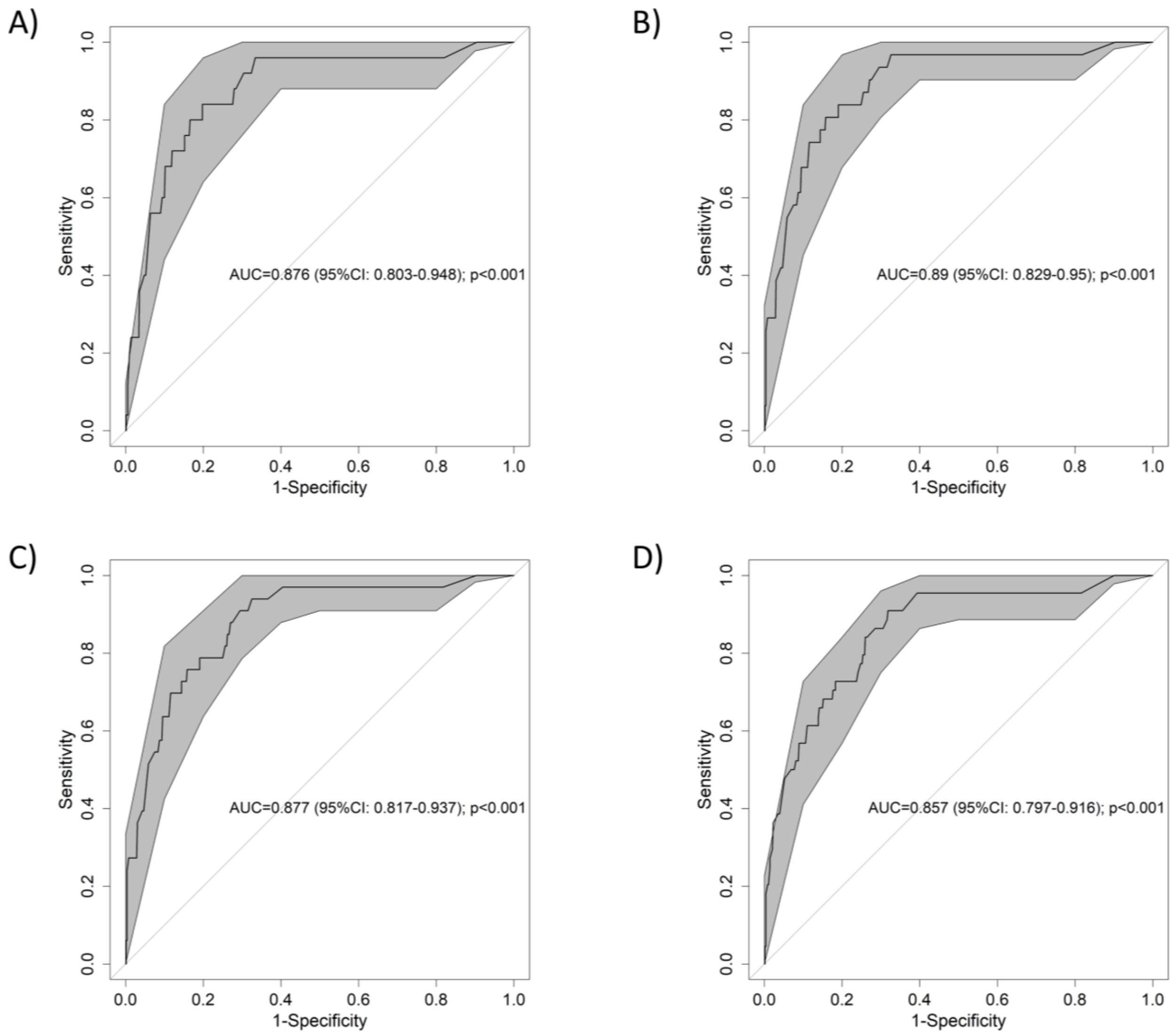


The bold line shows the ROC curve value, and the grey shading is the result of the validation coefficient. In the center of the graph is the area under the curve (AUC) and its 95% confidence interval.

© 2021 Martín-Rodríguez F et al. *JAMA Network Open.*

## **eAppendix.** Statistical Power Calculation

The statistical power (from 1 to 100) of the present study considering the sample used for training (3,081 patients), a significant level of p=0.001, and a SaFi difference between survivors and non survivors of 90 units (standard deviation = 20) is equal to 100.

© 2021 Martín-Rodríguez F et al. *JAMA Network Open.*
